# Pathophysiology, clinical manifestations, and prognostic insights of cerebral fat embolism: a literature review

**DOI:** 10.3389/fneur.2025.1732428

**Published:** 2026-01-12

**Authors:** Abdulrahim Saleh Alrasheed, Amna Mutasim Elazrag, Mohammad Salem Alqahtani, Faisal Alabbas

**Affiliations:** 1 Department of Neurosurgery, College of Medicine, King Faisal University, Al Ahsa, Saudi Arabia; 2 Faculty of Medicine, University of Khartoum, Khartoum, Sudan; 3 Neurosurgery Department, King Fahad Hospital of the University, Imam Abdulrahman Bin Faisal University, Al Khobar, Saudi Arabia

**Keywords:** cerebral fat embolism, embolism, fat embolism, neuroimaging, review, thromboembolism

## Abstract

Cerebral fat embolism (CFE) syndrome, a rare and often incomplete variant of fat embolism syndrome (FES), is characterized by pure neurological involvement and may occur after both traumatic and nontraumatic events. It is typically caused by embolization of fat droplets into the systemic circulation, most commonly after orthopedic trauma. The clinical presentation ranges from subtle neurological symptoms to life-threatening events, including respiratory distress, altered mental status, seizures, and coma. Diagnosis is challenging due to nonspecific signs, concurrent injuries, and lack of definitive diagnostic criteria. Although the Gurd and Wilson and Modified Gurd criteria are widely used, they are not fully validated and may not capture the full clinical picture. Neuroimaging, particularly MRI, plays a key supportive role. Despite investigations into corticosteroids and other pharmacologic agents, no treatment has demonstrated definitive efficacy. Thus, management remains largely supportive. Therefore, prompt recognition, refinement of diagnostic criteria, and further research into reliable biomarkers and targeted therapies are essential for improving CFE outcomes.

## 1 Introduction

Cerebral fat embolism (CFE) syndrome is an incomplete form of fat embolism syndrome (FES) characterized by isolated cerebral involvement without significant respiratory or dermatological signs (1, 2). The incidence of CFE is reported to be between 0.9 and 11% among patients with long bone fractures (3). It typically presents within 12–72 h following trauma, especially in multiple pelvic bone fractures or bilateral long bone fractures (1).

Despite its clinical significance, CFE poses significant challenges due to its potential rapid neurological deterioration (1). Furthermore, there are no universally accepted diagnostic criteria for CFE (4). While MRI has emerged as a promising supportive tool, its findings often lack specificity (1). Additionally, CFE is associated with a high mortality rate, and no specific therapy has been established to date (3).

This literature review aims to provide a comprehensive analysis of CFE. By synthesizing current evidence, we aim to enhance understanding, promote early diagnosis, and guide effective management strategies.

## 2 Methods

A comprehensive literature search was performed across multiple electronic databases, including PubMed, Web of Science, Scopus, and the Cochrane Library, up to April 2025. The search strategy employed a combination of keywords and MeSH terms such as “fat embolism,” “cerebral fat embolism,” “neurological symptoms,” “MRI,” “starfield pattern,” “diagnosis,” “biomarkers,” “treatment,” and “prognosis.”

All identified articles were initially screened by title and abstract independently by two reviewers. Articles considered potentially relevant underwent full-text review. Any discrepancies were resolved by consensus. Findings from the selected literature were synthesized descriptively and organized into major diagnostic and clinical domains. Due to the heterogeneity of available data and the narrative nature of this review, no formal quality assessment or quantitative synthesis was performed.

## 3 Epidemiology and risk factors

### 3.1 Epidemiology

Although subclinical fat globules are found in 82%−95% of trauma-related deaths (5), FES incidence rate varies widely, with reports of 0.008%−7.9% across all fractures (6, 7). FES typically occurs between 10 and 40 years old (7, 8). The higher incidence in young patients may stem from the increased fragility of older individuals, for whom minimal energy is needed to cause fracture (3). Therefore, older individuals are more likely to sustain simpler, low-energy fractures that do not facilitate high-fat droplet dissemination. CFE incidence rates following long bone fractures range from 0.9 to 11% (3), with higher rates after orthopedic procedures (8, 9). These findings highlight the variability in reported rates, likely reflecting differences in diagnostic criteria, study populations, and assessment methods. Intraoperative echocardiographic monitoring identifies embolic phenomena in 41%−87% of fixation procedures (10, 11). Additionally, peripheral sampling near fracture sites finds fat globules in 67%−95% of cases (12).

Male sex has been reported as a risk factor for FES development (3, 7, 8). It is attributed to greater high-impact trauma exposure, bone marrow composition differences, or hormonal factors (3, 13). However, data on sex differences are conflicting, with some studies suggesting higher susceptibility in males, while others report no significant gender disparity (1). Recently, the incidence rates fell substantially due to improvements in early fracture stabilization and surgical techniques (10, 14).

### 3.2 Risk factors

FES and CFE commonly follow trauma, particularly orthopedic injuries involving long bones such as the femur, tibia, and pelvis (15, 16). Their incidence increases with fracture number, location, severity, intramedullary nailing, and joint arthroplasty, reflecting a direct relationship between skeletal injury and embolic load (8, 15, 16). Delayed surgical fixation also increases the risk, emphasizing the importance of timely orthopedic intervention (4, 17, 18). Additionally, soft tissue trauma, including crush injuries, can trigger FES through marrow fat mobilization and systemic inflammatory responses (15, 16). While the incidence of FES is significantly low in cases of multiple soft tissue injuries, the fatality rate is very high (15).

Non-traumatic factors can precipitate FES. These include metabolic disturbances (e.g., Diabetes mellitus, acute pancreatitis, and fatty liver disease), cardiovascular–renal syndromes, systemic conditions associated (e.g., Sepsis, burns, and decompression sickness), and surgical procedures (e.g., Cardiopulmonary bypass, bone marrow transplantation (8, 19), and some cosmetic procedures) (20) (Table 1).

**Table 1:** Risk factors for FES and CFE.

**Category**	**Risk factors**
Traumatic	Long bone fractures (femur, tibia, and pelvis), multiple fractures, soft tissue crush injuries, and burns [8, 15–18]
Orthopedic	Intramedullary nailing, joint arthroplasty, and delayed fracture fixation [8, 15, 16]
Metabolic	Acute pancreatitis, fatty liver disease, diabetes mellitus, and decompression sickness [8, 19]
Other surgical/medical	Cardiopulmonary bypass, bone marrow transplantation, liposuction, and parenteral lipid infusions [8, 19, 20]
Patient-specific	Age < 30 years, male sex, PFO (20%−25% prevalence), and concurrent pulmonary embolism [3, 19]

PFO, patent foramen ovale.

## 4 CFE pathophysiology

FES and CFE are believed to result from a combination of mechanical and biochemical processes. The mechanical theory posits that trauma, particularly long bone fractures and orthopedic interventions, leads to the direct entry of fat globules from bone marrow or adipose tissue into torn venous channels and marrow sinusoids, which are richly interconnected with the vascular system (14, 21). These fat globules, often ranging from 7–10 μm in diameter, can traverse the venous circulation and lodge in the pulmonary capillaries. In certain scenarios, such as the presence of a right-to-left cardiac shunt (e.g., PFO) fat globules may bypass the pulmonary filter and reach the systemic arterial circulation, including cerebral vessels, leading to CFE (1, 19, 21). Intraoperative transesophageal echocardiogram (TEE) studies have detected fat embolization in 41% of patients during the fixation of long-bone fractures (14).

Migration is more likely when fat emboli are numerous (>100 particles/mm^2^) and sufficiently small; larger particles (>20 μm) are typically trapped within the pulmonary vasculature. Notably, CFE may occur without preceding respiratory symptoms, either due to direct cerebral passage of microemboli or sufficient pulmonary reserve (22).

The biochemical theory emphasizes the role of trauma-induced systemic inflammation and metabolic dysregulation. Stress from injury leads to catecholamine release, which mobilizes fat stores. Lipase and other enzymes break down triglycerides into free fatty acids (FFAs) and glycerol (4, 6). FFAs are cytotoxic and disrupt endothelial integrity, increasing capillary permeability, initiating edema, and triggering inflammatory cascades involving interleukins (e.g., IL-6) and tumor necrosis factor-α (1, 19, 23) Fat emboli activate the coagulation cascade, leading to microthrombi that exacerbate cerebral ischemia and hypoperfusion (4). These effects underlie multi-organ damage, including pulmonary, neurologic, and dermatologic manifestations (24). This explains non-traumatic FES/CFE, where systemic inflammation, capillary fragility, and fat mobilization occur without direct skeletal injury (1, 10).

## 5 Clinical presentation

Traditionally, FES presents with a classic triad of respiratory failure, neurological dysfunction, and a petechial rash within 24–72 h following triggering events (10, 25). However, this triad is observed only in < 10% of cases, contributing to under-recognition in clinical settings (26). Initial presentation may involve non-specific symptoms such as low-grade fever, tachycardia, or mild confusion, often preceding more overt respiratory or neurological signs (25, 27).

Respiratory symptoms are the earliest and most common, occurring in up to 75% of patients (21, 28). Early signs include tachypnea, dyspnea, and mild hypoxemia. In more severe cases, progression to acute respiratory distress syndrome (ARDS) may occur (25, 27). 44% of the patients with FES require mechanical ventilation (4). These findings correlate with fat microembolization in pulmonary capillaries and the associated inflammatory response.

The petechial rash occurs in 33% of patients (4), most commonly affecting the conjunctivae, axillae, oral mucosa, upper chest, and neck. It typically appears within 24–72 h and usually resolves spontaneously within a few days (15).

Neurological symptoms occur in up to 86% of FES cases and may develop within hours to days after the initial insult (29). The neurological symptoms generally follow a predictable clinical course, which can aid in timely diagnosis and management. The earliest manifestations include confusion, stupor, and lethargy (2). As the embolic process progresses, these symptoms may deepen into severe neurological deterioration, manifesting as coma, seizures, or localized neurological deficits such as aphasia or hemiplegia (3). Long-term cognitive impairment is frequently reported, with affected individuals experiencing memory disturbances and reduced attention span (30). This sequential progression underscores the need for vigilant neurological assessment in trauma patients at risk for FES.

CFE can present with paroxysmal sympathetic hyperactivity (PSH), characterized by episodic hypertension, tachycardia, hyperthermia, diaphoresis, and abnormal posturing. PSH is believed to result from autonomic center dysregulation in thalamic and brainstem multifocal lesions (31, 32). Its presence serves as a potential diagnostic clue, and it may guide targeted supportive measures (31). Additionally, severe cerebral edema and refractory intracranial pressure hypertension have been observed (33). Oliguria and jaundice have been documented also (34).

## 6 Diagnosis of CFE

Diagnosing FES and CFE remains clinically challenging due to the absence of universally accepted clinical criteria. Symptom onset varies widely, and initial manifestations can be non-specific, thus further complicating early diagnosis (4). Therefore, a high index of suspicion, comprehensive clinical evaluation, and timely imaging are critical, especially in polytrauma or post-surgical patients.

Gurd and Wilson's criteria are the most widely used diagnostic framework. The modified Gurd's criteria include additional neuroimaging features reflecting the potential cerebral compromise. However, they lack specificity and have not been fully validated (35). Schonfeld's FES index assigns numerical values to various signs, with a score of ≥5 indicating FES. Although it offers a semi-quantitative approach, it is rarely applied in CFE due to its lack of neurological weighting (36). Lindeque's criteria focus purely on respiratory compromise and are better suited for pulmonary-dominant FES rather than isolated cerebral involvement (37). While not diagnostic, the Glasgow coma scale (GCS) remains useful for assessing consciousness and neurological function in suspected CFE, but it lacks specificity in distinguishing CFE from other encephalopathies (38). Overall, although these frameworks do not capture the full clinical spectrum of CFE, underscoring the need for newer diagnostic systems (Tables 2–4).

**Table 2:** Original and modified Gurd's FES diagnostic criteria.

**Criteria**	**Guard and Wilson's criteria [35]**	**Modified Gurd's criteria [35]**
FES diagnosis threshold	Two major or one major + four minor features are required	One major + three minor or two major + two minor features are required
Major criteria	Petechiae	Petechiae on the conjunctiva and upper trunk
	Hypoxemia	PaO_2_ < 60 mmHg at FiO_2_ 0.2, with or without pulmonary infiltrate on X-ray
	Altered mentality	Altered mentality with multiple cerebral white matter lesions on brain MRI
Minor criteria	Tachycardia	HR > 110 bpm
	Fever	Fever >38 °C
	Unexplained anemia	Anemia with coagulopathy or DIC without a definite bleeding site
	Thrombocytopenia	Platelet count < 100^9^ × 10^3^/μl
	Retinal embolism	Retinal embolism
	Anuria or oliguria	Anuria or oliguria
	Fat globule in urine/sputum	–
	High ESR	–
	Jaundice	–

DIC, disseminated intravascular coagulation; ESR, erythrocyte sedimentation rate; FES, fat embolism syndrome; HR, heart rate; PaO_2_, arterial oxygen partial pressure.

**Table 3:** Schonfeld's criteria for FES diagnosis.

**Schonfeld's criteria (36)**	**Score**
≥**5 points are required**	
Petechiae	5
X-ray infiltrate on chest (diffuse alveolar infiltrate)	4
Hypoxemia	3
Mental confusion	1
Tachycardia	1
Fever	1
Tachypnea	1

**Table 4:** Lindeque's criteria for FES diagnosis.

**Lindeque's criteria (37)**
Presence of all features is required
Sustained PaO_2_ < 60 mmHg
Sustained PaCO_2_ >55 mmHg
RR >35/min despite adequate sedation
Increased work of breathing (dyspnea, tachycardia, or anxiety)

PaO_2_, arterial oxygen partial pressure; PaCO_2_, arterial carbon dioxide partial pressure; RR, respiratory rate.

Laboratory biomarkers are used as supportive diagnostic clues in FES/CFE. Common laboratory findings include thrombocytopenia and anemia (25). Arterial blood gas (ABG) analysis often reveals hypoxemia and respiratory acidosis, consistent with pulmonary involvement (39). Detection of fat globules in peripheral smears stained with Oil Red O stains has shown diagnostic value in ~79%−93% of trauma patients (40). Bronchoalveolar lavage can detect fat-laden macrophages within the first 24 h (39, 41, 42). However, it is a non-specific finding that can be seen in cases of sepsis, severe hyperlipemia, and propofol infusion (39, 41).

An isolated case report has described fat detection utility through pulmonary artery catheterization (15). Although most of the literature focused on fat droplet detection as a diagnostic clue, the absence of fat globules, especially in urine, does not rule out FES. All of these findings are nonspecific for CFE (39). S100B, a neuronal injury marker, may serve as an indicator of CFE (43, 44). However, this biomarker is non-specific for CFE and can be present in traumatic brain injuries (45).

### 6.1 Diagnostic imaging

In FES, imaging of the lungs is often nonspecific. Chest X-rays may show bilateral patchy infiltrates that resemble ARDS (46). Ventilation/perfusion (V/Q) scans may demonstrate scattered, patchy sub-segmental perfusion deficits with regular ventilation patterns (4).

**Figure 1:**
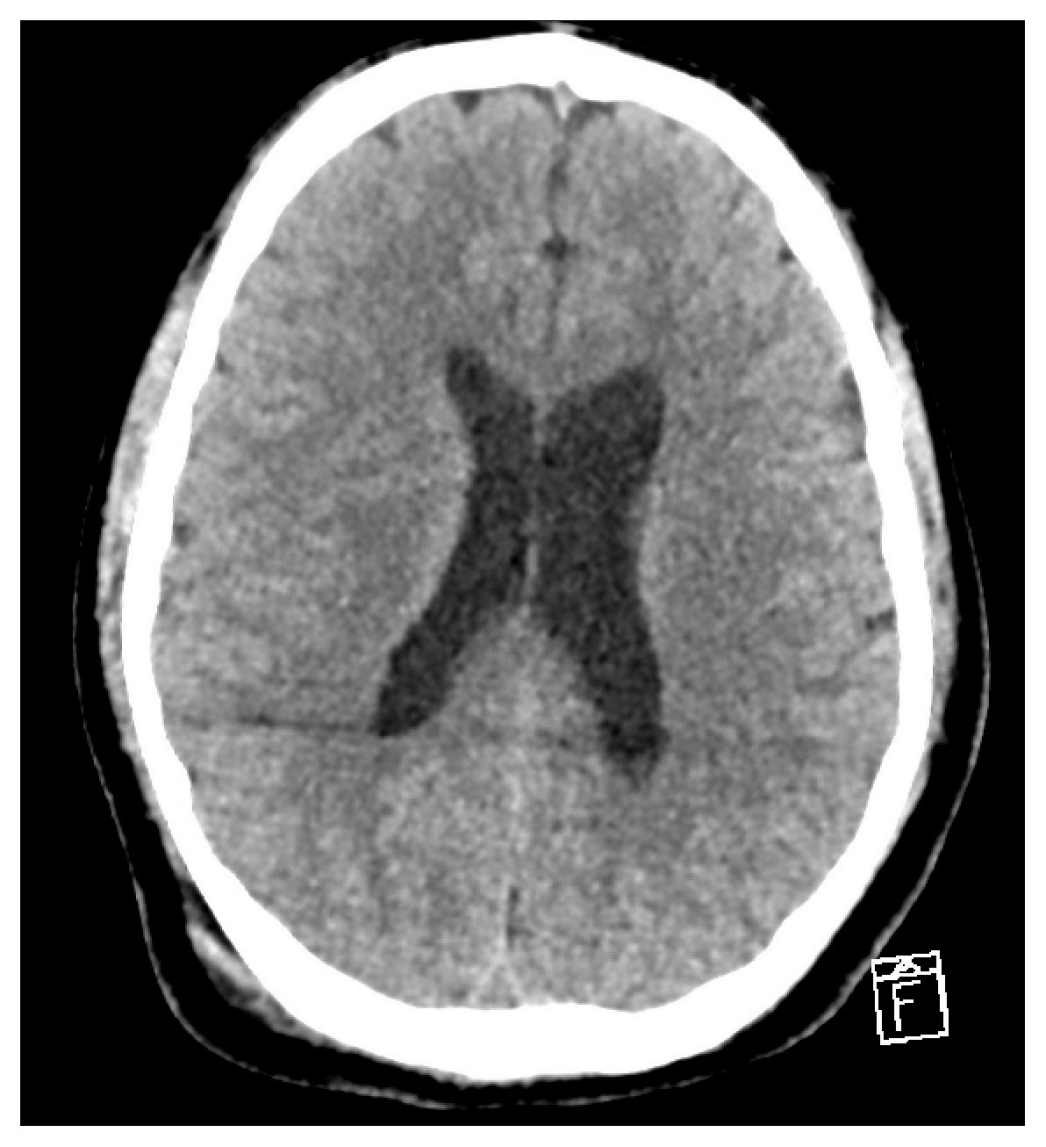
Axial non-contrast brain CT showing normal attenuation of the cerebral parenchyma with reserved gray-white matter differentiation. No mass effect or midline shift is noted.

Brain computed tomography (CT) is usually normal in CFE (47) (Figure 1). When CT scans are abnormal, they are typically nonspecific and may include diffuse cerebral edema or small hypodense lesions, most commonly in the periventricular white matter (48, 49). CT is typically recognized as less sensitive and specific than MRI in detecting CFE, and it is frequently used to rule out other potential causes of neurologic impairment (50). However, in severe or delayed presentations, CT may reveal scattered hypodense circular lesions with fat density, strongly supporting the diagnosis of CFE (1).

One notable CT finding is the hypodense artery sign, often observed following cardiac surgery or long bone fractures, characterized by a visibly low attenuation of a cerebral artery [most commonly the middle cerebral artery (MCA)]. It suggests the presence of macroscopic emboli with fat particle density (51). Although both air and fat may appear completely black on routine brain window settings, they can be reliably differentiated using quantitative CT attenuation measurements. Air on CT has an extremely low attenuation (approximately -1,000 HU), whereas fat demonstrates attenuation values ranging from −50 to −100 HU (52, 53). In contrast, the hyperdense artery sign typically indicates acute thrombotic occlusion and appears attenuated in the proximal MCA. Thus, a hypodense artery sign may indicate intravascular fat embolism, whereas a hyperdense artery sign is more commonly associated with acute thrombotic occlusion (54).

**Figure 2:**
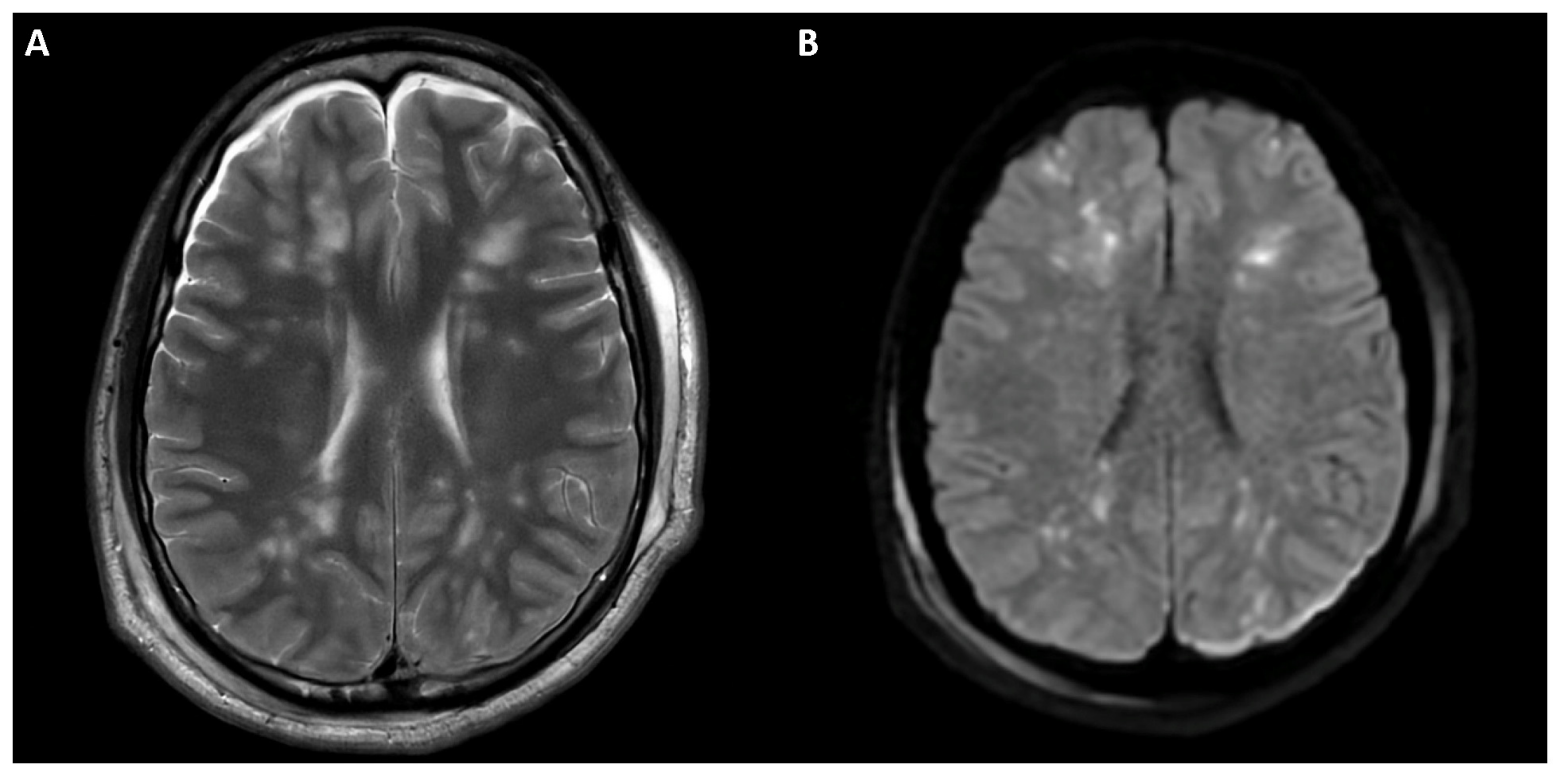
Axial brain T2/FLAIR MRI showing punctate, scattered hyperintense foci in the subcortical bilateral frontoparietal white matter area with relative cortical sparing and no significant mass effect **(A)**. Corresponding DWI demonstrating multiple punctate diffusion restriction foci consistent with CFE **(B)**.

In the CFE acute stage, MRI, especially diffusion-weighted imaging (DWI), shows the characteristic “starfield pattern,” a pattern of multiple small foci with restricted diffusion in white matter, centrum semiovale, thalami, basal ganglia, cerebellum, and the brainstem, usually around the watershed zones of the major cerebral arteries. On DWI, these lesions appear hyperintense, whereas on T2WI, they are iso to hyperintense (Figure 2). Confluent and cytotoxic edema, primarily affecting the corpus callosum, posterior internal capsule, cerebellar peduncles, and periventricular and subcortical white matter, is observed in the subacute stage. Differential diagnosis includes hypoglycemic encephalopathy, post-anoxic injury, and toxic leukoencephalopathy. Vasogenic edema may appear as a small dot or patch shaped T2/FLAIR hyperintensity without DWI restriction and can show contrast enhancement, indicating transient BBB disruption (1, 55).

Gliosis, cerebral atrophy, and chronic demyelination may be observed during the chronic phase. They present as small, T2 hyperintense dot-like lesions characterized by increased diffusion (1, 55). Susceptibility-weighted imaging (SWI) may reveal petechial hemorrhages by visualizing blood products, iron content, and small vascular structures through all CFE phases (1, 55–57). They usually manifest in the perivascular spaces of both white and deep gray matter, commonly affecting the cerebellum, brainstem, and corpus callosum. However, similar imaging findings can also be observed in diffuse axonal injury (DAI). On T2WI, CFE lesions are more numerous and scattered, while DAI lesions are fewer, larger, and more linear at the gray-white junction and within the corpus callosum. On DWI, CFE manifests as diffuse, confluent regions of restricted diffusion, whereas DAI is characterized by a few scattered foci (58).

### 6.2 Supportive diagnostic tools

TEE can effectively identify right-to-left shunting and should be considered, especially in patients with unexplained or disproportionate neurological findings (59). Transcranial Doppler (TCD) ultrasound is a valuable bedside tool for detecting microembolic signals (MES) in real time. MES appear as high-intensity transient signals (HITS) lasting < 300 ms and they are unidirectional, high-amplitude, and accompanied by a characteristic “chirping” sound. Some patients show only a few signals; others exhibit a high MES burden, reflecting a greater embolic load (60). MES is highest in the first 36 h following trauma; however, MES may be detectable up to 10 days after the incident (61). MCA mean flow velocity of 160 cm/s is considered indicative of severe hyperemia associated with CFE (62).

Electroencephalography (EEG) is useful in CFE to evaluate patients with altered mental status, especially when seizures are present (63). Digital subtraction angiography (DSA) can reveal distal vascular occlusions in cases of extensive fat embolization (64). Fundoscopic examination may show retinal signs suggestive of embolic events, including cotton wool spots, fat globules in retinal vessels, or retinal hemorrhages (65).

## 7 Preventive strategies

Prevention of CFE begins with early control of risk factors, particularly within the first 24 h after trauma. It is critical in patients with long bone or pelvic fractures. Early orthopedic stabilization reduces the duration of ongoing bone marrow embolization into the systemic circulation. Techniques that limit intramedullary pressure are strongly recommended. These include the use of smaller-diameter intramedullary nails or unreamed nailing. Thereby, reducing the volume of embolized fat and the risk of CFE (1, 4, 66).

Anatomical right-to-left shunts, such as PFO, can enable fat emboli to bypass the pulmonary capillary filtration system and directly enter the systemic arterial circulation, reaching the cerebral vasculature. In selected patients, particularly those with recurrent paradoxical embolic events, percutaneous closure of the PFO may work as a preventive strategy, reducing the embolic load and the intensity of the emboli. However, the supporting evidence remains lacking, compromising the real clinical significance of this measure (67).

Corticosteroids (CCS) possess anti-inflammatory and membrane-stabilizing properties, potentially reducing endothelial injury, capillary leakage, and secondary cerebral or pulmonary edema (4). While several studies have demonstrated a preventive effect in high-risk trauma patients, their role in reducing mortality remains inconclusive. Some authors advocate for early administration of intravenous dexamethasone as both a prophylactic and therapeutic measure, though evidence is still evolving (68, 69). Prophylactic anticoagulation administration is critical for preventing venous thromboembolism in immobilized patients. Low molecular weight heparin or unfractionated heparin is commonly used in this setting (1, 4).

## 8 Acute management

### 8.1 Systemic management

CFE management remains largely supportive, as no specific pharmacological therapy can reverse fat embolization (39, 70). Sodium dehydrocholate has been theorized to emulsify circulating fat globules, but its use remains largely experimental. Low molecular weight dextran is thought to improve microvascular flow and reduce blood viscosity; however, its administration carries the risk of renal dysfunction and coagulopathy. Albumin binds free fatty acids and may reduce their cytotoxicity. A 5% alcohol-glucose solution has been hypothesized to disperse fat emboli, though no clinical benefit has been demonstrated to date (1).

Early supplemental oxygen therapy is critical to improve oxygen delivery to tissues. In patients who exhibit significant respiratory compromise, mechanical ventilation may be required (71). In cases that progress to acute respiratory distress syndrome (ARDS), lung-protective ventilation strategies, including low tidal volume ventilation ( ≤ 6 ml/kg), titrated positive end-expiratory pressure (PEEP) starting from 12 to 15 cm H_2_O, and prone positioning, are recommended to minimize ventilator-induced lung injury and optimize gas exchange (39).

If hypoxemia remains refractory despite conventional support, escalation to extracorporeal membrane oxygenation (ECMO) should be considered. ECMO has shown a favorable impact on prognosis in FES, particularly in cases of severe collapse (39). In patients presenting with seizures, antiepileptic drugs should be administered (1).

Fat emboli in the pulmonary circulation may elevate pulmonary artery pressures, increase right ventricular (RV) afterload and result in RV strain or failure. Management includes optimizing oxygen delivery, providing inotropic support with agents such as dobutamine and isotonic fluids, and using selective pulmonary vasodilators like inhaled nitric oxide to reduce pulmonary vascular resistance. In more severe cases, especially when RV failure coexists with refractory hypoxemia, ECMO may be required for hemodynamic stabilization (39).

### 8.2 Acute neurological management

Sedation can help minimize agitation and control sympathetic overactivity (1, 39). Hyperbaric oxygen therapy (HBOT) enhances oxygen delivery to ischemic cerebral tissues and increases the blood oxygen pressure. HBOT has been associated with improved neurological outcomes in CFE (1, 27). Despite its potential, the limited availability of hyperbaric facilities and lack of high-quality randomized trials currently limit its widespread adoption.

In patients who develop severe ischemic stroke or malignant edema following fat emboli, neurological deficits may be more pronounced than in typical FES. Therefore, decompressive craniectomy may be required (72, 73). Endovascular clot retrieval (EVT) is emerging as a promising treatment modality, using techniques such as stent-retrievers and aspiration catheters. For successful EVT, several factors should be considered, including occlusion site, surgical anatomy, operator preference, and clot composition. Thrombolysis may be less effective for fat or calcified emboli, so a lower threshold for EVT is generally recommended (74). Therefore, personalized management strategies should be implemented based on the clinical context.

## 9 Prognosis

CFE carries a favorable recovery profile (1). However, the prognostic profile should be considered in the context of concomitant diseases. The reported mortality rate for CFE ranges between 5 and 15%, with a notable proportion of deaths attributable to respiratory failure (47). Favorable outcomes are more likely when brain MRI shows the classic reversible “starfield” pattern (70). Nonetheless, some patients may develop persistent cognitive impairments, including memory problems or executive dysfunction, especially if there was delayed treatment or prolonged hypoxia (30, 39). Status epilepticus, though relatively uncommon, has been described in CFE and may signal a more severe disease course (1).

Neuroimaging findings, especially the distribution and burden of lesions, strongly correlate with clinical outcomes. Poor recovery is more likely when lesions are extensively distributed throughout the cerebral hemispheres (1). In survivors of severe CFE, chronic brain atrophy has been reported as a delayed complication, particularly in those who experienced diffuse cerebral swelling in the acute phase (32). Unfortunately, due to the limited evidence available regarding CFE, there are no solid evidence-based features that can be used to reliably predict outcomes.

## 10 Limitations

This review has several limitations that should be acknowledged. The lack of standardized diagnostic protocols and variable use of advanced imaging and laboratory tests complicate direct comparison between management protocols. Limited data exist on the long-term neurocognitive and functional outcomes of CFE survivors, and few studies systematically evaluate the correlation between lesion characteristics and prognosis. The impact of coexisting medical conditions, differences in treatment approaches, and the potential significance of new biomarkers are not yet fully understood and may represent cofounding factors. To address these challenges and strengthen the evidence informing clinical care, multicenter prospective studies using standardized diagnostic criteria and thorough outcome evaluations are critically needed.

## 11 Conclusion

CFE is a rare and potentially fatal subtype of FES. It represents a diagnostic challenge due to the inconsistent presentation, lack of standardized criteria, and overlapped characteristics with other neurological disorders. MRI, especially DWI, serves as a sensitive diagnostic technique. The concurrent presence of a “starfield pattern” and orthopedic trauma serves as a supportive diagnostic clue. To date, there is no proven effective therapy. Management is purely supportive. Preventive measures, such as early fracture stabilization and other cautious intraoperative manipulation, are recommended. Early recognition, respiratory support, and neuroprotective strategies are essential to optimizing outcomes. Despite some patients achieving full recovery, others may suffer long-term neurological sequelae. This review highlights the urgent need for unified diagnostic criteria, improved prognostic markers, and prospective studies to improve the detection and management of both FES and CFE.
